# Water Costs of Gas Exchange by a Speckled Cockroach and a Darkling Beetle

**DOI:** 10.3390/insects11090632

**Published:** 2020-09-14

**Authors:** Waseem Abbas, Philip C. Withers, Theodore A. Evans

**Affiliations:** School of Biological Sciences, University of Western Australia, Crawley, Western Australia 6009, Australia; philip.withers@uwa.edu.au (P.C.W.); theo.evans@uwa.edu.au (T.A.E.)

**Keywords:** water loss, respiratory, metabolic rate, continuous gas exchange, discontinuous gas exchange, cuticular permeability

## Abstract

**Simple Summary:**

Evaporative water loss during metabolic gas exchange is an unavoidable cost of living for terrestrial insects. This respiratory water cost of gas exchange (the ratio of respiratory water loss to metabolic rate) is suggested to depend on several factors, such as the mode of gas exchange (convection vs. diffusion), species habitat, body size and measurement conditions. We measured this cost for a blaberid cockroach and a tenebrionid beetle using flow-through respirometry. We controlled the factors that affect respiratory water cost of gas exchange, i.e., both species are similar in their mode of gas exchange (dominantly convective), habitat (relatively moist) and body size, and were measured at the same temperature. The cockroaches showed both continuous and discontinuous gas exchange patterns, which had a significantly different metabolic rate and respiratory water loss but the same respiratory water cost of gas exchange. The darkling beetles showed a continuous gas exchange pattern only, and their metabolic rate, respiratory water loss and respiratory water cost of gas exchange were equivalent to those cockroaches using continuous gas exchange. This finding from our study highlights that the respiratory water cost of gas exchange is similar between species, regardless of the gas exchange pattern used, when the confounding factors affecting this cost are controlled. However, the total evaporative water cost of gas exchange is much higher than the respiratory cost because cuticular water loss contributes considerably more to the overall evaporative water loss than respiratory water. We suggest that the total water cost of gas exchange is likely to be a more useful indicator of species distribution with respect to environmental aridity than just the respiratory water cost.

**Abstract:**

Respiratory water loss during metabolic gas exchange is an unavoidable cost of living for terrestrial insects. It has been suggested to depend on several factors, such as the mode of gas exchange (convective vs. diffusive), species habitat (aridity), body size and measurement conditions (temperature). We measured this cost in terms of respiratory water loss relative to metabolic rate (respiratory water cost of gas exchange; RWL/${\dot{V}}_{CO_{2}}$) for adults of two insect species, the speckled cockroach (*Nauphoeta cinerea*) and the darkling beetle (*Zophobas morio*), which are similar in their mode of gas exchange (dominantly convective), habitat (mesic), body size and measurement conditions, by measuring gas exchange patterns using flow-through respirometry. The speckled cockroaches showed both continuous and discontinuous gas exchange patterns, which had significantly a different metabolic rate and respiratory water loss but the same respiratory water cost of gas exchange. The darkling beetles showed continuous gas exchange pattern only, and their metabolic rate, respiratory water loss and respiratory cost of gas exchange were equivalent to those cockroaches using continuous gas exchange. This outcome from our study highlights that the respiratory water cost of gas exchange is similar between species, regardless of gas exchange pattern used, when the confounding factors affecting this cost are controlled. However, the total evaporative water cost of gas exchange is much higher than the respiratory cost because cuticular water loss contributes considerably more to the overall evaporative water loss than respiratory water. We suggest that the total water cost of gas exchange is likely to be a more useful index of environmental adaptation (e.g., aridity) than just the respiratory water cost.

## 1. Introduction

Gas exchange by terrestrial insects takes place through the tracheal system. This system extends from spiracles on the exterior of the body, and inside the body as a network of air-filled tubules that branch further to become thin-walled tracheoles, which are in close proximity to tissues and are the site of gas exchange [1,2]. Spiracles open to allow the diffusion of oxygen from the atmosphere into, and carbon dioxide out of, the insect’s body. However, water vapor moves out with CO_2_, as atmospheric air is usually drier than the air in trachea. This respiratory water loss (RWL) during breathing is considered a cost of gas exchange [2,3].

Water balance is a substantial problem for terrestrial insects due to their small body size and consequent large surface area to volume ratio. RWL, as a cost of gas exchange, varies depending on ambient temperature and environmental water availability, and is therefore typically a higher portion of total water loss for insects living in arid compared to mesic environments. RWL can be reduced by closing the spiracles, and the dynamics of their opening and closing are responsible for gas exchange patterns [4,5]. Cuticular water loss (CWL) is a second, often substantial avenue for evaporative water loss that occurs continuously. It is quite variable between species, depending primarily on the aridity of the insect’s environment [6].

Insects show three distinct patterns of gas exchange, based on their modulation of spiracle opening: Continuous, cyclic and discontinuous gas exchange (CGE, Cyclic and DGE, respectively) [7,8]. CGE and cyclic are the ancestral patterns, as all spiracles do not seal completely or simultaneously during gas exchange. In CGE, the spiracles remain open and the gas exchange is regular, often to support maximum gas exchange in active species [8,9,10]. Cyclic gas exchange consists of alternating burst and interburst phases that produce periodic gas exchange, but the spiracles never close completely and simultaneously [11]. DGE is considered to be the most derived pattern, as it has periods of complete synchronous closure of all spiracles, and these periods may vary in duration according to environmental conditions [8,9,12].

DGE, the most complex pattern of gas exchange, has three distinct phases of spiracular activity: Closed (C), flutter (F) and burst (B). These are produced by modulation of spiracle closure based on partial pressure differences of both CO_2_ (*P*CO_2_) and O_2_ (*P*O_2_) between the ambient environment and the insect tracheal system. Spiracles remain closed during the C phase. The *P*O_2_ drops during the C phase due to metabolic consumption in tissues, whereas *P*CO_2_ rises as CO_2_ is produced and continues to dissolve in the haemolymph [2,13]. The C phase ends, and the F phase begins with rapid opening and closing of the spiracles, triggered by low (~4 kPa) intratracheal *P*O_2_ [14]. During the F phase, there is an influx of ambient O_2_ to maintain *P*O_2_ at a steady level (~2–4 kPa), whereas there is no CO_2_ loss against the bulk influx of O_2_, and *P*CO_2_ continues to rise [7]. The B phase begins when the spiracles open fully, to allow the maximum intake of O_2_ and release of CO_2_. The cycle starts again when intratracheal *P*CO_2_ reaches a threshold of ~4–6 kPa and the spiracles close [13,15,16]. DGE has received more attention than the other patterns, with ten hypotheses proposed to explain its evolutionary origins, of which eight are adaptive and two are nonadaptive, but no hypothesis has received unequivocal support [9,15].

The mode of spiracular gas exchange (convective vs. diffusive) is likely to play an important role in determining the relationship between metabolism and RWL, i.e., the respiratory water cost of gas exchange (RWL/${\dot{V}}_{CO_{2}}$) apart from any evolutionary significance of DGE for reducing RWL [2,17]. For example, Kestler [18] notes that insects can minimize RWL at any given metabolic rate using convective rather than diffusive gas exchange, regardless of which gas exchange pattern is used. Woods and Smith (2010, [17]) found support for their prediction that if the mode of gas exchange is the same for different taxonomic groups, then the scaling exponent for relationship between metabolic rate and RWL will be 1, i.e., a lower rate of metabolism is associated with a lower RWL and vice versa. The scaling relationship between metabolic rate and RWL is, in fact, the respiratory water cost of gas exchange. Besides the mode of gas exchange, other factors, such as environmental aridity and ambient temperature, affect the respiratory water cost of gas exchange [17]. However, comparisons between insect species of the respiratory water cost of gas exchange are lacking, especially when all the above described factors are considered. 

We compared the respiratory water costs of gas exchange for adults of two insect species from different orders, the speckled cockroach (*Nauphoeta cinerea* Olivier 1789; Blaberidae) and the darkling beetle (*Zophobas morio* Fabricius 1776; Tenebrionidae). We expected similar respiratory water costs of gas exchange for both species, since both species use a same dominantly convective mode of gas exchange (abdominal pumping, [19,20]), are mesic [21,22,23], are of comparable body mass, were raised under identical conditions and were measured at same temperature and time of day. Before comparing the respiratory water costs of gas exchange between both species, we first measured the temporal patterns for gas exchange, and instantaneous and average ${\dot{V}}_{CO_{2}}$ (a proxy for metabolic rate) and ${\dot{V}}_{H_{2}O}$ (total evaporative water loss) using flow-through respirometry.

## 2. Materials and Methods

### 2.1. Animals

Speckled cockroaches were purchased from Livefoods Unlimited (Adelaide, SA, Australia). They were maintained in a plastic container with a mesh lid, and the sides of the container were lined with Vaseline to prevent escape. Cockroaches were provided with dry cat food (Purina Fancy Feast, Rhodes, NSW, Australia) and empty cardboard egg trays provided harbourage. Water-filled glass vials with cotton wool in the open end provided drinking water. Darkling beetles were purchased from Bugs ‘N’ Things (Oldbury, WA, Australia). They were maintained in a plastic container covered with a cheese cloth lid. Bran and carrots were provided, along with drinking water. Cockroaches and beetles were maintained at a constant temperature of 25 °C ± 1 SE, 12 L:12 D photoperiod, ambient gaseous conditions (21% O_2_, 0.03% CO_2_) and ambient RH (range 60–80%).

### 2.2. Respirometry

CO_2_ emission and evaporative water loss were measured for 23 cockroaches and 8 beetles using standard flow-through respirometry. Only adult cockroaches and beetles were used. Individuals were selected for measurement randomly and irrespective of sex. Both species are active at night [24,25,26], and we measured them during their inactive state (daytime) to avoid the effect of activity on measurements. Each insect was weighed to 0.01 g with a digital balance (HL 200i A&D Company Limited, Toshima-Ku, Tokyo, Japan) before and after measurement. 

Each insect was measured individually in a respirometry chamber that was constructed from half of a 10-mL Terumo syringe barrel. Each respirometry measurement lasted for 3 h, and chambers were empty for the first and last 30 min of the experiment to obtain the baseline concentrations of each gas. We measured both species between 11:00 a.m. and 3:00 p.m. in a thermally controlled laboratory (23.1 °C ± 0.09 SE) to minimise any effect of temperature variation on gas exchange patterns. Individuals were observed to be immobile (‘at rest’) during the respirometry. 

Compressed air was passed through a column of sodasorb (W. R. Grace & Co., Chicago, IL, USA) to scrub CO_2_, and drierite (Hammond drierite, Xenia, OH, USA) to absorb moisture. Some cockroaches were measured using air from a compressed air cylinder (BOC Gases, Canning Vale, WA, Australia), which had low and constant background concentrations of CO_2_ and moisture and did not need to be scrubbed. The dry, CO_2_-free air flow entering the chamber was regulated at 25 mL min^−1^ STPD using a mass flow controller (AFC 2600 Aalborg, Orangeburg, NY, USA, or C22208 Sierra Instruments, Monterey, CA, USA). Mass flow controllers were calibrated with a Gilian Gilibrator 2 (Sensidyne, St. Petersburg, FL, USA). Excurrent air from the respirometry system entered a brass housing containing a temperature humidity probe (HMP113, Vaisala Corporation, Helsinki, Finland) to measure relative humidity (RH) and temperature of excurrent air (T_a_). The probe was calibrated using a DewPoint Generator DG-4 (Sable Systems International, Las Vegas, NV, USA) and a traceable mercury glass thermometer (Australian Calibrating Services, Melbourne, Vic, Australia). The CO_2_ concentration of excurrent air was then measured with a CO_2_ analyser (S151 Qubit systems, Kingston, Ontario, CA, USA). The CO_2_ analyser was calibrated using a certified span gas (0.153 ± 0.003 (SE)% CO_2_, BOC Gases, Canning Vale, WA, Australia).

### 2.3. Data Acquisition and Analysis

Digital voltmeters (Protek 506, Seoul, Korea and Thurlby 1905a, Thurlby Electronics Ltd., Huntingdon Cambridgeshire, UK) were used to measure analogue signals from the Vaisala probes and CO_2_ analysers, respectively. These voltmeters were connected via a USB port hub (UC2324, ATEN, North Ryde, NSW, Australia) to a desktop PC. Voltage signals were sampled every 10 s and converted to ppm for CO_2_ concentration, percent relative humidity for water vapour and degree Celsius for temperature using a custom written program (Visual Basic 6, written by PC Withers). Data were stored continuously in an Excel file during each respirometry trial. Acquired data were analysed with a laptop using an Excel spreadsheet (written by PC Withers and W Abbas). The raw values of CO_2_, H_2_O and temperature were first calibration corrected. The rate of CO_2_ emission (STPD ${\dot{V}}_{CO_{2}}$, mL g^−1^ h^−1^) was calculated based on the following equations from Withers (2001, [27]).

(1) $${\dot{V}}_{{\mathrm{CO}}_{2}}=[({V˙}_{e}\times F_{e}{\mathrm{CO}}_{2})-({V˙}_{I}\times F_{i}{\mathrm{CO}}_{2})]\times 60/m$$

where V̇_I_ is the incurrent flow rate (STPD mL min^−1^), V̇_e_ is the excurrent flow rate of air (STPD mL min^−1^), F_i_CO_2_ is the incurrent fraction of CO_2_, F_e_CO_2_ is the excurrent fraction of CO_2_ and m is the body mass (g). All these variables were measured or calculated, except V̇_e_, which was calculated as

V̇_e_ = V̇_I_ × ((F_i_CO_2_/RER) + F_i_O_2_ + (1 − F_i_O_2_ − F_i_CO_2_ − F_i_H_2_O)) ÷ (1 − F_e_H_2_O − F_e_CO_2_ + (F_e_CO_2_/RER)) (2)

where F_i_O_2_ is the incurrent fraction of O_2_, F_e_O_2_ is the excurrent fraction of O_2_, F_i_H_2_O is the incurrent fraction of H_2_O, F_e_H_2_O is the excurrent fraction of H_2_O and RER is the respiratory exchange ratio (assumed to be 0.85, [28]). The incurrent and excurrent fractions for water were calculated from T_a_ and RH using hygrometric equations [29].

The rate of evaporative water loss ${\dot{V}}_{H_{2}O}$ (mg g^−1^ h^−1^) was calculated as

(3) $${\dot{V}}_{H_{2}O}=((V˙{\text{’}}_{e}\times {\mathrm{WVD}}_{e})-(V˙{’}_{I}\times {\mathrm{WVD}}_{i}))\times 60/(m\times 1000)$$

where V̇’_I_ is the incurrent flow rate (ATP mL min^−1^), V̇’_e_ is the excurrent flow rate of air (ATP mL min^−1^) and WVD (subscripts e and i are for incurrent and excurrent, respectively) is the water vapour density (ATP mg L^−1^), calculated from T_a_ and RH using hygrometric equations [29]. The ${\dot{V}}_{CO_{2}}$ and ${\dot{V}}_{H_{2}O}$ values were then baseline-corrected assuming linear drift from start to end baseline. Instantaneous correction of ${\dot{V}}_{CO_{2}}$ and ${\dot{V}}_{H_{2}O}$ followed Bartholomew et al. (1981, [30]). The ${\dot{V}}_{CO_{2}}$ and ${\dot{V}}_{H_{2}O}$ values were then lag-corrected.

### 2.4. Gas Exchange Patterns

The ${\dot{V}}_{CO_{2}}$ trace for each insect was examined to characterise the respiratory pattern. An individual was considered to use DGE if its rate of CO_2_ emission had a clear closed phase (i.e., ${\dot{V}}_{CO_{2}}$ regularly at or close to zero) [31]. For calculations, both C and F phases were combined as the interburst phase (IB) due to poor distinction between them, following Wobschall and Hetz (2004, [32]). DGE cycles (*n* = 2–8 per individual insect) were analysed for each insect to calculate ${\dot{V}}_{CO_{2}}$ (mL g^−1^ h^−1^), and the duration (sec) for each phase and the entire cycle (IB + B). DGE cycles were analysed for calculation of water loss rate (${\dot{V}}_{H_{2}O}$, water loss peaks corresponded with simultaneous CO_2_ emission) as for ${\dot{V}}_{CO_{2}}$. For insects showing the CGE pattern, ${\dot{V}}_{CO_{2}}$ and ${\dot{V}}_{H_{2}O}$ were calculated as averages over time.

We partitioned evaporative water loss during the B phase of DGE into its cuticular (CWL) and respiratory (RWL) components after Hadley and Quinlan (1993, [33]). This is now termed the ‘traditional method’ [34]. CWL was calculated as the average water loss during the IB phase, and RWL was calculated from the average during the B/F phases by subtracting the CWL. For insects using CGE, CWL (μg h^−1^) and RWL (μg h^−1^) were calculated by the linear regression method [35], where CWL is the intercept of the regression equation, and RWL was calculated from the slope of the equation (R; µg H_2_O µL CO_2_^−1^), which is the ratio of RWL/ ${\dot{V}}_{CO_{2}}$.

(4) $${\dot{V}}_{H_{2}O}=\mathrm{CWL}+(R\times {\dot{V}}_{{CO}_{2}})$$

Cuticular permeability (CP, µg h^−1^ cm^−2^ hPa^−1^), the CWL (µg h^−1^) expressed per unit surface area (SA; cm^2^) per saturation deficit (WVPD; hPa) was calculated after Appel et al., (1983, [21]) as

CP = CWL/(SA × WVPD)(5)

The surface area of cockroaches was calculated using Meeh’s formula as cited by Gray and Chown (2008, [34]) as

SA = 12 × body mass(g)^0.63^ (6)

where the constant is 12 based on several arthropod measurements [34,36]. The saturation deficit, the driving force for evaporative water loss through the cuticle, was calculated as the difference between the water vapour pressure at ambient relative humidity and temperature and the saturated water vapour pressure at the same temperature [21,37]. For insects showing DGE, water vapour pressure was calculated using RH during the closed phase (to reflect CWL conditions). For insects using CGE, which were analysed using the regression method, uncorrected CP (CWL = ${\dot{V}}_{H_{2}O}$; including RWL) was calculated first and then corrected by subtracting RWL following Schilman et al. (2005, [38]) and Rolandi et al. (2014, [39]). 

To compare the RWL cost of metabolic rate between patterns of two species, metabolic rate (after conversion to ${\dot{V}}_{O_{2}}$ mol day^−1^ using RER = 0.85) and RWL (${\dot{V}}_{H_{2}O}$ mol day^−1^) were regressed against each other.

### 2.5. Interspecific Comparisons

To compare the metabolic cost associated with gas exchange patterns, the metabolic rate and body mass of species of cockroaches (order: Blattodea) and of beetles (family: Tenebrionidae) were compiled from the published literature. For comparison, all metabolic rates were converted to 23 °C equivalence using a Q_10_ of 2.0, which is a consensus value for insects [40]. The metabolic rate (mL h^−1^; log transformed) was regressed against body mass (g; log transformed) separately for CGE and DGE gas exchange patterns of cockroaches and of beetles. To fit the respiratory water cost of gas exchange for our species to the Woods and Smith [17] model, a total of 30 insect species from Woods and Smith [17] were used to determine the relationship between RWL and oxygen uptake rate, with confidence and prediction intervals.

### 2.6. Statistical Analyses

Means are presented with standard error (s.e.m) and sample size (*n*). Physiological variables metabolic rate (${\dot{V}}_{CO_{2}}$, (µL g^−1^ h^−1^), total evaporative water loss (${\dot{V}}_{H_{2}O}$, µg g^−1^ h^−1^), CWL (µg g^−1^ h^−1^), RWL (µg g^−1^ h^−1^), % CWL (percent fraction of total evaporative water loss), % RWL (percent fraction of total evaporative water loss) and CP (µg h^−1^ cm^−2^ hPa^−1^) of cockroaches showing CGE were compared with DGE cockroaches and with beetles showing CGE using general linear models in R (lm function; v. 4.0.0; R Development Core Team, 2020, [41]). RWL and ${\dot{V}}_{O_{2}}$ were log transformed and scaled against each other using linear regressions for individual cockroaches showing CGE and DGE and beetles showing CGE, and then linear regressions of these two variables were compared between two species for the respiratory water cost of gas exchange. Comparisons of linear regressions were performed using StatistiXL (www.statistical.com) version 2.0 and α was 0.05 for all tests.

## 3. Results

**Figure 1:**
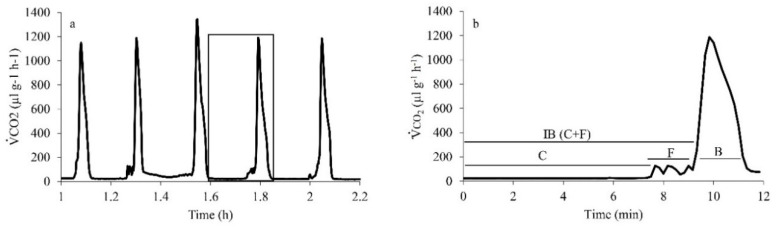
Example of discontinuous gas exchange (DGE) trace for the speckled cockroach. The one DGE cycle in the rectangle in (**a**) is enlarged in (**b**). C is for closed phase, F for flutter, IB is for interburst phase (C and F combined) and B is for burst phase.

**Figure 2:**
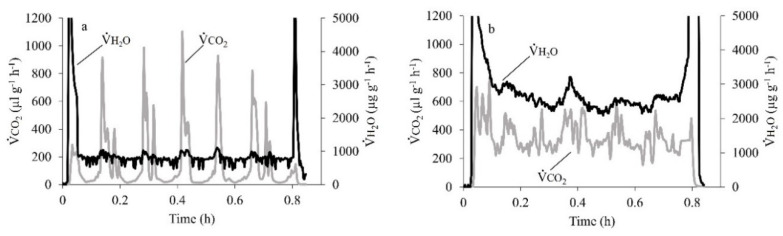
Metabolic rate, ${\dot{V}}_{CO_{2}}$ (µL g^−1^ h^−1^), and total evaporative water loss, ${\dot{V}}_{H_{2}O}$ (µg g^−1^ h^−1^), measured over time for an individual cockroach showing the discontinuous gas exchange (DGE) pattern (**a**) and for an individual beetle showing continuous gas exchange (CGE) pattern (**b**).

Of the 23 cockroaches measured, 15 used DGE (675 ± 37 mg, *n* = 15), with the reminder using CGE (808 ± 54 mg, *n* = 8). None of the individuals switched between CGE and DGE during the 2-h respirometry duration. The cockroach DGE pattern clearly showed the three phases C, F (combined as Interburst, IB) and B (Figure 1). Both IB (177 ± 35 s) and B (200 ± 21 s) phases contributed almost equally (44% and 56%, respectively) to the total DGE duration (377 ± 44 s). However, the release of CO_2_ was considerably higher during the B phase (90%) than IB (10%, Table 1). Beetles used only used CGE (580 ± 16 mg, *n* = 8). There was a clear correspondence of ${\dot{V}}_{H_{2}O}$ and ${\dot{V}}_{CO_{2}}$ patterns for cockroaches breathing discontinuously and a lesser correspondence for a beetle breathing continuously (Figure 2).

Within the same species, the metabolic rate of cockroaches showing DGE (195 ± 8.2 µL g^−1^ h^−1^) was 25% lower than CGE cockroaches (262 ± 33 µL g^−1^ h^−1^) and differed significantly (t_28_ = 2.20, *p* = 0.037). RWL, as a fraction of the total water loss, was significantly lower for cockroaches showing DGE than CGE (DGE: 10.8 ± 1.3%; CGE: 17.3 ± 3.2%; t_28_ = 2.43, *p* = 0.022), hence % CWL was significantly higher for cockroaches showing DGE than CGE (DGE: 89.2 ± 1.31%; CGE: 82.7 ± 3.15%; t_28_ = 2.43, *p* = 0.022). There were no significant differences for any other physiological variables between CGE- and DGE-breathing cockroaches (total evaporative water loss ${\dot{V}}_{H_{2}O}$ (µg g^−1^ h^−1^): t_28_ = 0.52, *p* = 0.61; RWL (µg g^−1^ h^−1^): t_28_ = 1.53, *p* = 0.14; CWL (µg g^−1^ h^−1^): t_28_ = 0.26, *p* = 0.80; CP (µg h^−1^ cm^−2^ hPa^−1^): t_28_ = 0.69, *p* = 0.50; Table 1).

At the interspecific level, cockroaches using CGE (808 ± 54 mg) were 28% heavier than CGE-breathing beetles (580 ± 16 mg; t_28_ = 3.37, *p* = 0.002; Table 1). Contrary to the intraspecific comparisons of cockroaches, metabolic rate and RWL as a fraction of total water loss did not differ significantly between cockroaches and beetles showing CGE (Mr: t_28_ = 1.27, *p* = 0.22; RWL%: t_28_ = 0.06, *p* = 0.95). Moreover, there were no significant differences in any physiological variables for cockroaches and beetles using CGE (total evaporative water loss, ${\dot{V}}_{H_{2}O}$ (µg g^−1^ h^−1^): t_28_ = 1.74, *p* = 0.09; RWL (µg g^−1^ h^−1^): t_28_ = 1.67, *p* = 0.11; CWL (µg g^−1^ h^−1^): t_28_ = 1.60, *p* = 0.12; CWL%: t_28_ = 0.06, *p* = 0.95; CP (µg h^−1^ cm^−2^ hPa^−1^): t_28_ = 1.58, *p* = 0.12; Table 1).

### 3.1. Metabolic Effect of Gas Exchange Patterns

**Figure 3:**
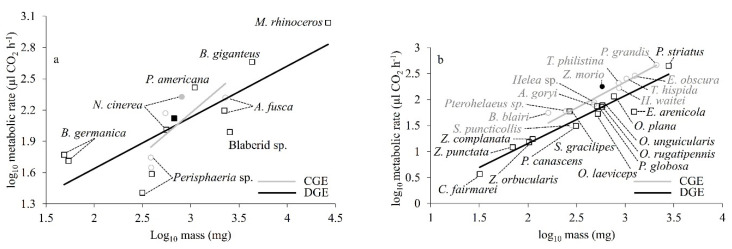
The relationships of log_10_ metabolic rate and log_10_ mass of (**a**) cockroach species, and (**b**) tenebrionid beetle species using either CGE or DGE. Data were corrected to a common measurement temperature of 23 °C, assuming a Q_10_ value of value of 2.0. Filled symbols are for data points from our study and open symbols are for data points from the literature (circles, CGE; squares, DGE; see Supplementary Tables S3 and S4 for further information).

Interspecific comparison of metabolic rate for CGE and DGE gas exchange patterns of cockroaches based on regression of log_10_MR and log_10_M showed that species that used either or both CGE and DGE patterns had a common allometric slope (0.548, *p* = 0.60) and intercept (−0.854, *p* = 0.89). That is, there was no difference in metabolic rate for cockroaches using either CGE or DGE (Figure 3a). However, the pattern for CGE was based on just two species, and therefore may not be meaningful. On the other hand, comparison of the relationships between logMR and logM of gas exchange patterns of tenebrionid beetles showed that CGE and DGE beetles had a common slope (0.940, *p* = 0.646) but a significantly different intercept (*p* = 0.001). In other words, beetles that used DGE had a lower metabolic rate than CGE beetles (Figure 3b).

### 3.2. Respiratory Water Cost of Gas Exchange

**Figure 4:**
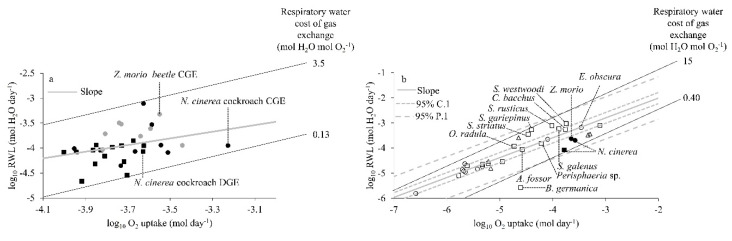
The relationships of log_10_RWL and log_10_ ${\dot{V}}_{O_{2}}$ for cockroach and beetle individuals from (**a**) our study, and (**b**) of 30 insect species showing different gas exchange patterns (CGE: Open circles; DGE: Open squares; cyclic: Open triangles; cockroach and beetle species are labelled) from Woods and Smith’s [17] model of respiratory water cost of gas exchange (see Supplementary Table S5). Three data points (filled symbols) for average respiratory water cost of gas exchange of individuals from our study were superimposed to this multispecies relationship for comparison. Higher and lower respiratory water costs of gas exchange are shown on both panels based on data spread.

When compared, the linear regression relationships of log_10_RWL (mol H_2_O day^−1^) and log ${\dot{V}}_{O_{2}}$ (mol O_2_ day^−1^) of the individual speckled cockroaches using CGE or DGE and beetles using CGE resulted in a common slope (0.263, *p* = 0.950), which indicates a constant respiratory water cost of gas exchange of about 0.76 mol H_2_O mol O_2_^−1^ (Figure 4a). There was considerable variation between individuals (0.13–3.5 mol H_2_O mol O_2_^−1^). The intercept was not significantly different between cockroach individuals using CGE or DGE (*p* = 0.374) and between cockroach and beetle individuals using CGE (*p* = 0.284). In other words, the respiratory water cost of gas exchange was similar within the same species for its individuals using different gas exchange patterns and between both species showing the same gas exchange pattern (Figure 4a).

The interspecific comparison of the respiratory water cost of gas exchange shows that DGE and CGE cockroaches and CGE beetles from our study fell with in the lower bound of the 95% prediction interval of Woods and Smith’s [17] multispecies model for relationship of log_10_RWL (mol H_2_O day^−1^) and logO_2_ uptake (mol O_2_ day^−1^). The positions of CGE and DGE cockroaches and CGE beetles in the multispecies relationship suggest that both species conform to the proposed model for respiratory water cost of gas exchange (Figure 4b). There was considerable variation between species (0.40–15 mol H_2_O mol O_2_^−1^).

## 4. Discussion

We found that speckled cockroaches maintained under the same housing conditions used either CGE or DGE patterns of gas exchange. Around two-thirds (65%) of cockroaches measured used DGE (*n* = 15), with the remainder using CGE (*n* = 8). This is consistent with a previous study of this species [42]. Similar intraspecies variation in gas exchange patterns has been reported for other cockroach species (Family Blaberidae), such as the Table Mountain cockroach (*Aptera fusca*) and *Persiphaeria* sp. [8,28]. The darkling beetle, with a similar body mass to cockroaches and maintained under the same housing conditions as speckled cockroaches, only used CGE and did not differ for metabolic rate, RWL or any other physiological variable from CGE cockroaches. There are no previous studies of this species, but our results are consistent with other related desert darkling beetles living under xeric conditions, such as *Eloedes obscura*, *Helea* sp. and *Brises blari* [43,44], that only use CGE. Both the speckled cockroach and beetle from our study were mesic and of similar body size, and used identical mode of gas exchange (convective, i.e., abdominal pumping). The similarity of these confounding factors is key for our comparison of the respiratory water cost of gas exchange, since we can interpret the similar cost between both species, and between CGE and DGE, to reflect a fundamental similarity in the physics of spiracular gas exchange which otherwise affects the respiratory water cost of gas exchange [17].

### 4.1. Metabolic Rate and Water Loss

The metabolic rate of DGE speckled cockroaches (195 µL g^−1^ h^−1^) is 56% higher than a previous measurement (125 µL g^−1^ h^−1^) for this species at same temperature [13]. This could be related to feeding status prior to measurement as Schimpf et al. [13] measured cockroaches after 24 hr of fasting, whereas we did not food-deprive cockroaches, and fasting has been found to downregulate metabolic rate for this species [31]. Similar fasting effects have been recorded for other species, such as ants [45], yellow mealworms (*Tenebrio molitor*) and lesser mealworms (*Alphitobius diaperinus*) [46]. The metabolic rate of CGE speckled cockroaches from an earlier study (270 µL g^−1^ h^−1^, adjusted to 23 °C assuming a Q_10_ value of 2, [20]), is comparable to that observed here (262 µL g^−1^ h^−1^). This was significantly higher than for DGE speckled cockroaches. 

The metabolic rate has not previously been measured for darkling beetles, but our value for *Zophobas morio* conforms well to the relationship between body mass and metabolic rate for other tenebrionid beetle species using CGE (Figure 3b). This substantiates metabolic rate for this species, being consistent with other tenebrionid beetle species of the comparable body mass. 

The total evaporative water loss rates of speckled cockroaches using CGE (0.998 mg h^−1^) or DGE (0.635 mg h^−1^) are within the range (0.5–2 mg h^−1^) reported by Schimpf et al. [31,47] for this species. Cockroaches using CGE were significantly heavier than those using DGE, suggesting heavier cockroaches were less constrained by water. 

### 4.2. Use of Qubit S151 Analyser to Characterise Gas Exchange Patterns

Gas analysers (typically CO_2_) used for flow-through respirometry with insects vary widely in their measurement range and precision. Due to their small size (body mass: 1 mg–4 g), smaller fluxes of CO_2_ from insects ideally require a higher measurement precision than measurement of larger animals (>10 g; e.g., Withers and Cooper, 2011) [48] because the flow rate cannot be reduced proportionally to lower metabolic rate (to maintain similar % O_2_ or CO_2_ levels in the excurrent air) for small animals. Insect respirometry studies often use high-precision CO_2_ analysers (e.g., LI-COR models 6251, 6262, 820, 7000; precision: ± 0.3–1 ppm) to characterise gas exchange patterns [49], but we used a low-cost respiration package (Qubit S151, precision: ±1 ppm) to characterise gas exchange patterns and metabolic rates for speckled cockroaches and darkling beetles. The Qubit S151 (precision: ±1 ppm) analyser is used commonly for respirometry with large animals, such as amphibians and reptiles [50,51], but it has been used for insects heavier than about 1 g [52,53,54]. We successfully used the low-cost Qubit S151 to characterise gas exchange patterns and metabolic rates for speckled cockroaches and darkling beetles (mass range: 500−850 mg). It remains to be seen how much smaller an insect in size could be measured for its gas exchange patterns using Qubit S151 analyser.

### 4.3. Metabolic Correlates with Gas Exchange Patterns

The metabolic rate and RWL, as a fraction of total evaporative water loss, differed for speckled cockroaches using CGE and DGE, whereas there was no significant difference between speckled cockroaches and darkling beetles using CGE (Table 1). It is important to note that we measured the speckled cockroaches and darkling beetles kept under the same housing conditions (ad lib water and food, and high ambient RH) and experimental conditions to characterise the gas exchange patterns and measure ${\dot{V}}_{CO_{2}}$ and ${\dot{V}}_{H_{2}O}$. There was no opportunity for short- or long-term acclimation to different ambient conditions (other than about 2 h in dry air for respirometric measurement). Consequently, our comparisons of patterns and measurements were not confounded by differences arising from acclimaation to differing housing/experimental conditions (e.g., water or food restriction, changes in RH or other gases; see Terblanche et al., 2010 [55], for the effects of acute and chronic acclimation/plasticity).

At the interspecific level (Figure 3a), the metabolic rate of cockroach species using CGE was not lower than the ones using DGE. However, this comparison may be problematic for two reasons. First, species (*n* = 3) using CGE are underrepresented compared to those using DGE (*n* = 8). Second, there might be phylogenetic nonindependence of species belonging to different families [56]. In contrast, the comparison of metabolic rates for beetle species using CGE and DGE (Figure 3b) found a difference in intercept (CGE had a higher metabolic rate) but there was a common allometric slope (${\dot{V}}_{CO_{2}}$ α M^0.94^). The significant metabolic rate difference for beetle species between CGE and DGE might reflect the larger sample sizes for the beetle comparison, and all of the beetle species belong to the same family (Tenebrionidae), so the phylogenetic effect may be small. However, an important further caveat for interpretation of these interspecies patterns is that all species are not likely to have been acclimated to the same environmental conditions and food and water regimes and may differ in adaptation to their natural environments. It is likely that all of these possible differences (and more) influence metabolic rate, along with gas exchange pattern. Detailed studies of the plasticity of insect gas exchange patterns are rare apart from a single study on acute and chronic oxygen and moisture effect [13] and acute and chronic temperature effects [55].

### 4.4. Respiratory Water Cost of Gas Exchange

The respiratory water cost of gas exchange (RWL/${\dot{V}}_{O_{2}}$; mol H_2_O mol O_2_^−1^) did not differ significantly for the speckled cockroaches using CGE and DGE, or the darkling beetles using CGE (Figure 4a). The slope of this relationship, or the respiratory water cost of gas exchange, was 0.76 mol H_2_O mol O_2_^−1^. It is important to appreciate that this similarity of respiratory water cost of gas exchange for different patterns (CGE, DGE) and species is expected, so long as the diffusive or convective mechanisms for respiratory H_2_O and O_2_ exchange are the same. The values of both species for the respiratory water cost of gas exchange also conformed to the multispecies model of Woods and Smith [17]. It is interesting to note that both these species lie in the lower bound of the prediction interval, suggesting a relatively lower respiratory water cost of gas exchange than other cockroach and beetle species included in the model (Figure 4b). 

We can extend Woods and Smith’s [17] concept of hygric cost of gas exchange from a respiratory water cost (mol RWL/mol ${\dot{V}}_{O_{2}}$) to an overall water cost (mol ${\dot{V}}_{H_{2}O}$/mol ${\dot{V}}_{O_{2}}$), which is a broader index of water economy reflecting environmental adaptations rather than physiological mechanisms. The overall water cost of gas exchange will be higher than the respiratory costs because of the additional CWL contribution to total WL. For the speckled cockroach from our study, the overall and respiratory water costs of gas exchange differ by ten times (6.0, 0.60 respectively) from each other for DGE and by six times (5.3, 0.90) for CGE individuals, indicating better overall water economy of DGE individuals. The overall and respiratory water costs of gas exchange differ by thirteen (41, 3.0) and by twelve times (32, 2.7) from each other for DGE and CGE individuals respectively of the Table Mountain cockroach [28]. The overall and respiratory water costs of gas exchange differ by five times (18, 3.44) for the *Perisphaeria* sp. cockroach [34].

At the level of interspecific comparisons, the overall water cost of gas exchange provides an index of the relative magnitude of cuticular permeability to water loss, which generally reflects the aridity of the species’ habitat and CWL [38,57]. For example, the overall and respiratory water costs of gas exchange of *Perisphaeria* sp. differs by five times compared to ten times for the speckled and thirteen times for the Table Mountain cockroaches, reflecting the more arid habitat of *Perisphaeria* sp. compared to the more mesic habitat of the speckled and Table Mountain cockroaches [28,34]. These considerable differences between respiratory and overall water costs of gas exchange point toward role of cuticular permeability, which directly affects relative contribution of CWL to overall evaporative water loss based on the aridity of species’ habitat.

### 4.5. Cuticular Permeability

Cuticular permeability (CP) is a useful measure of water loss because it expresses the rate of cuticular water loss relative to the driving force for evaporation and body surface area. The CP for the speckled cockroaches using CGE (5.5 ± 1.8 µg h^−1^ cm^−2^ hPa^−1^) based on the regression method is similar to the value from the direct method for the individuals showing DGE (4.48 ± 0.44 µg h^−1^ cm^−2^ hPa^−1^). The similar results from both these methods is consistent with an earlier study for a closely related blaberid cockroach, *Perisphaeria* sp., having CP calculated using both methods [34]. Together, these results increase the credibility of use of the regression method for the CP calculation of individuals using CGE. Further, the CP of the speckled cockroach individuals showing DGE from our study is comparable to CP of DGE individuals of the closely related blaberid species, *Perisphaeria* sp. (6.49 µg h^−1^ cm^−2^ hPa^−1^, [34]), and the German cockroach (7.42 µg h^−1^ cm^−2^ hPa^−1^, [58]). These estimates for CP were all determined using flow-through respirometry, and are more accurate measures of CP than other methods derived from gravimetric measurements using weight loss over time to calculate total water loss.

These estimates for CP were determined using flow-through respirometry, and they differ from CP determined by gravimetric measurement. Gravimetric CP uses weight loss over time to calculate total water loss and is considerably higher for cockroaches and beetles [59,60]. The gravimetric approach may include weight loss from faeces, urine and metabolism/respiration, and even damage to the cuticle by handling and abrasion during handling [61,62]. Also, gravimetric CP determined from dead individuals may be increased due to a loss of spiracular control [58,60]. Thus, we support the findings of other studies [28,34,58] that the respirometric measurement of CP is more accurate than the gravimetric measurement of CP.

### 4.6. Interburst Phase Release of CO_2_: Cuticular or Spiracular?

During the interburst phase of DGE, spiracles are generally considered to be completely closed. However, for some insects from various orders, there is a measurable release of CO_2_ during this phase. The speckled cockroach from our study releases 10% of its overall cycle ${\dot{V}}_{CO_{2}}$ during the IB phase, reflecting a relatively low cuticular ${\dot{V}}_{CO_{2}}$ but over a much longer IB phase than B phase. A blaberid cockroach releases around 8% of its overall ${\dot{V}}_{CO_{2}}$ during the IB phase [8], the German cockroach (*Blatella germanica*) about 6% [58], *Perisphaeria* sp. about 15% [63] and the American cockroach (*Periplaneta Americana*) about 27% [64]. The desert locust *Schistocerca gregaria* releases about 10% of its total ${\dot{V}}_{CO_{2}}$ during the IB phase [65] and a desert ant species (*Cataglyphis bicolor*) releases about 14% of the total CO_2_ release [66].

The presence of this small (in terms of ${\dot{V}}_{CO_{2}}$ rate) but often substantial (in terms of IB phase duration) cuticular release of CO_2_ has not attracted much attention in the literature. There is no consensus on whether this CO_2_ release is spiracular [33], cuticular [67,68] or both [69,70]. A lack of coordination of closure for spiracles, such that some are partially open even during the IB phase and release CO_2_, is a possible source of IB ${\dot{V}}_{CO_{2}}$ [69]. For example, insects that have many spiracles, such as the fruit fly and German cockroach, may not coordinate the closure of all spiracles at the same time [58,69,70]. Alternatively, spiracular release during the IB phase could result from independent functioning of the spiracles from each other, causing some spiracles to be open in this phase [71,72]. Wasserthal [73] provided support for the IB spiracular release through the direct visualization of thoracic spiracles of resting blow flies (*Calliphora vicina*) while simultaneously measuring CO_2_ release. Alternatively, some studies [67,68] have suggested that CO_2_ might diffuse across the cuticle (along with water) during the IB phase. Unequivocal universal support for either trans-spiracular or trans-cuticular release is lacking at present.

## 5. Conclusions

Our study of speckled cockroaches and darkling beetles, maintained under similar conditions, indicated a significantly lower metabolic rate and RWL as a fraction of total water loss of cockroaches showing DGE than CGE, which is consistent with hygric hypothesis. Allometric analyses for metabolic patterns with body mass found no difference for cockroach species using CGE or DGE (probably reflecting the small data set for cockroaches), whereas beetle species using CGE had a higher metabolic rate than those using DGE (but the same allometric scaling). However, interspecies analyses such as this might confound other factors (starvation, dehydration, acclimation/acclimatisation, environmental adaptations) with gas exchange pattern. The respiratory water cost of gas exchange did not differ between CGE and DGE, or speckled cockroaches and darkling beetles as we expected, but the overall water cost of gas exchange water loss for gas exchange was considerably higher, reflecting the substantial CWL compared to RWL. We suggest that overall water cost of gas exchange could provide a broader index of water economy that includes species differences in cuticular permeability, hence hygric adaptations to the species’ environmental aridity.

## Figures and Tables

**Table 1:** Gas exchange characteristics of cockroaches showing DGE and CGE and beetles showing CGE at an average temperature of 23 °C and at a flow rate of 25 mL min^−1^.

Species	(*N. cinerea*)		(*Z. morio*)
Pattern	DGE (*n* = 15)	CGE (*n* = 8)	CGE (*n* = 8)
Mass (mg)	675 ± 36.9 *	808 ± 53.9	580 ± 16.4 *
Durations (seconds)			
Total	377 ± 42.6		
Interburst	177 ± 35.0		
Burst	200 ± 21.3		
Interburst%	44.1 ± 4.61		
Burst%	55.9 ± 4.61		
${\dot{V}}_{CO_{2}}$ (µL g^−1^ h^−1^)			
Interburst	42.1 ± 4.46		
Burst	341 ± 26.5		
Interburst%	9.99 ± 1.61		
Burst%	90.2 ± 2.77		
metabolic rate	195 ± 8.17 *	262 ± 33.1	307 ± 28.1 ^ns^
${\dot{V}}_{H_{2}O}$ (µg g^−1^ h^−1^)			
Interburst	849 ± 91.6		
Burst	1049 ± 104		
Interburst%	39.4 ± 4.16		
Burst%	61.0 ± 4.19		
TEWL	943 ± 93.9 ^ns^	1113 ± 367	1765 ± 285 ^ns^
CWL (µg g^−1^ h^−1^)	849 ± 91.6 ^ns^	924 ± 330	1456 ± 233 ^ns^
RWL (µg g^−1^ h^−1^)	93.7 ± 35.2 ^ns^	189 ± 76.4	309 ± 53.3 ^ns^
CWL (%)	89.2 ± 1.31 *	82.7 ± 3.15	82.5 ± 0.54 ^ns^
RWL (%)	10.8 ± 1.31 *	17.3 ± 3.15	17.5 ± 0.54 ^ns^
CP (µg h^−1^ cm^−2^ hPa^−1^)	4.38 ± 0.44 ^ns^	5.46 ± 1.78	8.31 ± 1.35 ^ns^

Body mass, metabolic rate and RWL (as a percentage of total water loss) were significantly different between cockroaches showing CGE and DGE (indicated by *), and all other variables were non-significant (indicated by ^ns^). Cockroaches and beetles showing CGE were not significantly different for all variables (indicated by ^ns^) except body mass (indicated by *). TEWL = total evaporative water loss, CWL = cuticular water loss, RWL = respiratory water loss, and CP = cuticular permeability; values are mean ± s.e.m (see Supplementary Tables S1 and S2 for summary).

## Supplementary Materials

The following are available online at https://www.mdpi.com/2075-4450/11/9/632/s1, Table S1: Summary of individuals of speckled cockroach (*n* = 15) that showed discontinuous gas exchange (DGE) used for calculation of mean values in main text (Table 1) of the measured physiological variables, Table S2: Summary of individuals of speckled cockroach (*n* = 8) and of beetles (*n* = 8) that showed continuous gas exchange (CGE) used for calculation of mean values in main text (Table 1) of the measured physiological variables, Table S3: Body mass and metabolic rate (both measured and Q_10_ corrected to 23 °C using a Q_10_ of 2) of cockroach species showing CGE and DGE from literature (8) and *Nauphoeta cinerea* from this study used for regression comparison of metabolic cost between CGE and DGE species shown in Figure 3a (columns shaded grey were used for regression relationships), Table S4: Body mass and metabolic rate (both measured and Q_10_ corrected to 23 °C using a Q_10_ of 2) of tenebrionid species showing CGE and DGE from literature (23) and *Zophobas morio* from this study used for regression comparison of metabolic cost between CGE and DGE species shown in Figure 3b (columns shaded grey were used for regression relationship), Table S5: Data for 30 insect species from Woods and Smith (2010) for the regression relationship of respiratory water cost of gas exchange, and the two species from our study were superimposed to the regression relationship (given at the end of the table),); 95% confidence and prediction intervals were calculated for the overall regression (Figure 4b). Shaded columns were used for regression relationship).
