# Supplementary material for: Water Costs of Gas Exchange by a Speckled Cockroach and a Darkling Beetle
Source: Insects. 2020 Sep 14;11(9):632. doi: 10.3390/insects11090632 (PMC7563770; doi:10.3390/insects11090632)
Supplement: Supplementary file 1 [file insects-11-00632-s001.pdf]

Table 1. Summary of individuals of speckled cockroach (n = 15) that showed discontinuous gas exchange (DGE) used for calculation of mean values in main text (Table 1) of the measured physiological variables. IB = Interburst; B = burst; TWL = total water loss; RWL = respiratory water loss; CWL = cuticular water loss; CP ($\mu g\ h^{-1}\ cm^{-2}\ hPa^{-1}$) = cuticular permeability.

	**VCO2**			**VCO2**		**Durations**			**Durations**		**VH2O**			**VH2O**		**TWL Components**				
**mg**	**$\mu L\ g^{-1}\ h^{-1}$**			**% of Total**		**seconds**			**% of Total**		**$\mu g\ g^{-1}\ h^{-1}$**			**% of Total**		**$\mu g\ g^{-1}\ h^{-1}$**		**% of Total**		
	**IB**	**B**	**total**	**IB**	**B**	**IB**	**B**	**overall**	**IB**	**B**	**IB**	**B**	**TWL**	**IB**	**B**	**RWL**	**CWL**	**RWL**	**CWL**	**CP**
500	39.2	442	158	17.2	85.6	612	270	882	69.4	30.6	1017	1422	1141	61.8	38.2	124	1017	10.9	89.1	5.17
970	43.5	327	153	19.5	68	215	100	315	68.3	31.7	858	1131	944	62	38	86.9	858	9.2	90.8	4.99
610	27.1	213	158	5.15	94.8	34.3	80	114	30	70	553	591	580	28.6	71.4	26.5	553	4.57	95.4	2.58
610	84.7	430	249	19.2	75.4	178	138	315	56.3	43.7	1198	1349	1264	53.4	46.6	65.9	1198	5.21	94.8	5.72
510	58.4	324	177	18.7	79	186	142	328	56.7	43.3	1266	1550	1389	51.7	48.3	123	1266	8.84	91.2	5.75
630	61.4	197	172	7.3	91	90	350	440	20.5	79.5	760	897	869	17.9	82.1	108	760	12.5	87.5	3.9
780	48.6	304	240	5.07	94.9	60	180	240	25	75	1446	1555	1528	23.7	76.3	82.1	1446	5.38	94.6	7.1
560	25.3	406	200	5.41	116	167	222	388	42.9	57.1	546	817	701	33.5	66.5	154	546	22	78	3
770	21	354	245	2.77	98	166	348	514	32.3	67.7	434	595	543	25.8	74.2	109	434	20	80	2.85
800	30.3	601	210	10.1	85.3	300	128	428	70.1	29.9	939	1381	1071	61.5	38.5	132	939	12.3	87.7	5.44
600	22.8	392	197	6.08	94.4	140	127	267	52.5	47.5	579	819	693	43.9	56.1	114	579	16.4	83.6	3.07
600	40.5	334	205	8.61	91.7	127	163	290	43.7	56.3	675	828	762	38.7	61.3	86.3	675	11.3	88.7	3.42
500	34.2	334	222	5.82	93.5	150	247	397	37.8	62.2	710	826	782	34.3	65.7	71.9	710	9.19	90.8	3.35
810	61.2	272	165	17.3	88	204	234	438	46.6	53.4	1478	1659	1575	43.7	56.3	96.9	1478	6.15	93.8	7.81
880	33.8	194	179	1.79	98.3	28	268	296	9.46	90.5	280	307	304	8.7	91.3	24.4	280	8.03	92	1.54

Table 2. Summary of individuals of speckled cockroach (n = 8) and of beetles (n = 8) that showed continuous gas exchange (CGE) used for calculation of mean values in main text (Table 1) of the measured physiological variables. TWL = total water loss; RWL = respiratory water loss; CWL = cuticular water loss; CP (µg h^{-1} cm^{-2} hPa^{-1}) = cuticular permeability.

Cockroach Individuals Showing CGE.								Beetle Individuals Showing CGE							
**Mass**	**VCO2**	**VH2O**	**TWL Components**					**Mass**	**VCO2**	**VH2O**	**TWL Components**				
**mg**	**µL g^{-1} h^{-1}**	**µg g^{-1} h^{-1}**	**µg g^{-1} h^{-1}**		**% of Total**			**mg**	**µL g^{-1} h^{-1}**	**µg g^{-1} h^{-1}**	**µg g^{-1} h^{-1}**		**% of Total**		
	**Total**	**TWL**	**RWL**	**CWL**	**RWL**	**CWL**	**CP**		**Total**	**TWL**	**RWL**	**CWL**	**RWL**	**CWL**	**CP**
990	475	708	85.6	612	270	882	3.86	520	278	2739	461	2278	16.8	83.2	12.1
840	175	446	68	215	100	315	2.41	580	255	2179	389	1790	17.8	82.2	10.3
1030	220	415	94.8	34.3	80	114	2.51	630	197	1493	230	1264	15.4	84.6	7.07
640	268	607	75.4	178	138	315	2.79	590	308	1105	220	885	19.9	80.1	5.42
750	326	609	79	186	142	328	2.77	610	469	789	139	650	17.6	82.4	3.96
870	236	3537	91	90	350	440	17.4	600	373	3073	595	2478	19.4	80.6	15.1
790	238	2024	94.9	60	180	240	9.41	490	246	826	143	683	17.3	82.7	3.46
550	161	561	116	167	222	388	2.53	620	327	1919	298	1621	15.5	84.5	9.1

Table 3. Body mass and metabolic rate (both measured and Q_{10} corrected to 23˚C using a Q_{10} of 2) of cockroach species showing CGE and DGE from literature (8) and *Nauphoeta cinerea* from this study used for regression comparison of metabolic cost between CGE and DGE species shown in Figure 3a (columns shaded grey were used for regression relationships).

Family	Species	Mass (M)	Pattern	Metabolic Rate (MR)	Measurement Temperature	MR (Q_{10}) at 23˚C	Log M	Log MR	References
		g		mL h^{-1}	˚C	mL h^{-1}			
Blaberidae	*Macropanesthia rhinoceros*	26.5	DGE	0.887	20	1.09	1.42	0.0382	[1]
	Blaberus giganteus	4.33	DGE	0.589	26.6	0.459	0.636	−0.338	[2]
	Blaberid sp	2.54	DGE	0.091	22	0.0975	0.405	−1.01	[3]
	Aptera fusca	2.29	CGE	0.12	15	0.209	0.359	−0.680	[4]
		2.23	DGE	0.09	15	0.157	0.348	−0.805	[4]
	Nauphoeta cinerea	0.808	CGE	0.212	23	0.212	−0.0929	−0.674	This study
		0.675	DGE	0.132	23	0.132	−0.171	−0.881	This study
		0.56	DGE	0.118	25	0.103	−0.252	−0.988	[5]
		0.547	CGE	0.170	25	0.148	−0.262	−0.829	[6]
	Perisphaeria sp.	0.394	DGE	0.0336	21	0.0386	−0.405	−1.41	[7]
		0.389	CGE	0.0362	20	0.0445	−0.410	−1.35	[8]
		0.386	CGE	0.0483	21	0.0555	−0.413	−1.26	[7]
		0.315	DGE	0.091	20	0.0255	−0.502	−1.59	[8]
Blattidae	*Periplaneta americana*	1.1	DGE	0.3	25	0.261	0.0414	−0.583	[9]
Blatellidae	*Blatella germanica*	0.054	DGE	0.021	10	0.0517	−1.27	−1.29	[10]
		0.049	DGE	0.024	10	0.0591	−1.31	−1.23	[10]

Table 4. Body mass and metabolic rate (both measured and Q_{10} corrected to 23˚C using a Q_{10} of 2) of tenebrionid species showing CGE and DGE from literature (23) and *Zophobas morio* from this study used for regression comparison of metabolic cost between CGE and DGE species shown in Figure 3b (columns shaded grey were used for regression relationship).

Species	Mass (M)	Pattern	Metabolic Rate (MR)	Measurement Temperature	MR (Q_{10}) at 23˚C	Log M	Log MR	References
	g		ml h⁻¹	˚C	ml h⁻¹			
Pimelia grandis Klug	2.10	CGE	0.535	25	0.466	0.322	−0.332	[11]
Eloedes obscura	1.26	CGE	0.27	22	0.289	0.0997	−0.539	[12]
Trachyderma hispida (Forskal)	1.03	CGE	0.292	25	0.254	0.0141	−0.595	[11]
Helea waitei Lea	0.865	CGE	0.192	25	0.167	−0.0630	−0.777	[13]
Trachyderma philistina Riche & Sauley	0.859	CGE	0.24	25	0.209	−0.0660	−0.680	[11]
Zophobas morio	0.58	CGE	0.178	23	0.178	−0.237	−0.750	This study
Helea sp.	0.481	CGE	0.102	25	0.0888	−0.318	−1.052	[13]
Akis goryi Solier	0.478	CGE	0.092	25	0.0801	−0.321	−1.10	[11]
Scaurus puncticollis Solier	0.282	CGE	0.037	25	0.032	−0.550	−1.49	[11]
Pterohelaeus sp. Brême	0.245	CGE	0.069	25	0.0601	−0.611	−1.22	[14]
Brises blairi Carter	0.162	CGE	0.064	25	0.0557	−0.790	−1.25	[13]
Psammodes striatus	2.8	DGE	0.364	20	0.448	0.447	−0.349	[15]
Epiphysa arenicola	1.24	DGE	0.0951	30	0.0585	0.0924	−1.23	[16]
Onymacris plana	0.767	DGE	0.188	30	0.115	−0.115	−0.937	[16]
O. unguicularis	0.585	DGE	0.127	30	0.0779	−0.233	−1.11	[16]
O. rugatipennis a.	0.573	DGE	0.117	30	0.0722	−0.242	−1.14	[16]
O. laeviceps	0.525	DGE	0.0867	30	0.0534	−0.280	−1.27	[16]
Physadesmia globosa	0.516	DGE	0.122	30	0.0748	−0.287	−1.13	[16]
Pimelia canascens	0.312	DGE	0.036	25	0.0313	−0.506	−1.50	[11]
Stenocara gracilipes	0.268	DGE	0.0965	30	0.0594	−0.572	−1.23	[16]
Zophosis complanata	0.112	DGE	0.02	25	0.0174	−0.951	−1.76	[11]
Zophosis orbicularis	0.103	DGE	0.0246	30	0.0151	−0.987	−1.82	[16]
Z. punctata	0.07	DGE	0.014	25	0.0122	−1.15	−1.91	[11]
Cardiosis fairmarei	0.032	DGE	0.006	30	0.00369	−1.49	−2.43	[16]

Table 5. Data for 30 insect species from Woods and Smith (2010) for the regression relationship of respiratory water cost of gas exchange, and the two species from our study were superimposed to the regression relationship (given at the end of the table); 95% confidence and prediction intervals were calculated for the overall regression (Figure 4b). Shaded columns were used for regression relationship).

Species	Mass	Gas Uptake Rate (X)	Water Loss Rate (Y)	Log X	Log Y	Predict	Confidence Interval		Prediction Interval		Pattern	References
	g	Mol O_2 Day^{-1}	Mol H_2O Day^{-1}			Y	Min	Max	Min	Max		
Perisphaeria sp	0.45	6.10E-05	1.54E-04	−4.21	−3.81	−3.76	−3.93	−3.59	−4.59	−2.93	CGE	[7]
Aphodius fossor	0.121	2.68E-05	8.85E-05	−4.57	−4.05	−4.04	−4.19	−3.89	−4.87	−3.22	DGE	[17]
Scarabaeus gariepinus	1.13	3.98E-05	5.44E-04	−4.40	−3.26	−3.91	−4.06	−3.75	−4.74	−3.08	DGE	[18]
Scarabaeus striatus	0.753	3.45E-05	3.56E-04	−4.46	−3.45	−3.96	−4.11	−3.80	−4.78	−3.13	DGE	[18]
Scarabaeus galenus	1.68	1.31E-04	5.81E-04	−3.88	−3.24	−3.50	−3.70	−3.29	−4.34	−2.66	DGE	[18]
Scarabaeus rusticus	1.06	9.70E-05	7.83E-04	−4.01	−3.11	−3.60	−3.79	−3.41	−4.44	−2.77	DGE	[18]
Scarabaeus westwoodi	1.76	1.81E-04	9.53E-04	−3.74	−3.02	−3.39	−3.61	−3.16	−4.23	−2.54	DGE	[18]
Circellium bacchus	7.19	1.76E-04	5.55E-04	−3.75	−3.26	−3.40	−3.61	−3.18	−4.24	−2.55	DGE	[19]
Eleodes obscura	1.28	3.48E-04	6.57E-04	−3.46	−3.18	−3.16	−3.42	−2.90	−4.01	−2.31	CGE	[12]
Omorgus radula	0.207	1.88E-05	1.19E-04	-4.73	−3.93	−4.17	−4.31	−4.0	−4.99	−3.34	DGE	[20]
Blattella germanica	0.0488	2.48E-05	2.67E-06	−4.61	−5.57	−4.07	−4.22	−3.92	−4.90	−3.24	DGE	[10]
Drosophila melanogaster	0.001	1.98E-06	1.39E-05	−5.70	−4.86	−4.94	−5.16	−4.72	−5.78	−4.10	CGE	[21]
Camponotus vicinus	0.0352	1.12E-05	2.87E-05	−4.95	−4.54	−4.34	−4.50	−4.19	−5.17	−3.51	DGE	[22]
Cataglyphis bicolor	0.0245	4.79E-06	2.27E-05	−5.32	−4.64	−4.64	−4.82	−4.46	−5.47	−3.80	DGE	[22]
Crematogaster californica	0.00136	3.80E-06	1.48E-05	−5.42	−4.83	−4.72	−4.91	−4.56	−5.55	−3.88	CGE	[23]
Dorymyrmex insanus	0.000579	2.24E-06	1.11E-05	−5.65	−4.96	−4.90	−5.11	−4.68	−5.74	−4.06	CGE	[23]
Forelius mccooki	0.00027	2.59E-07	1.55E-06	−6.59	−5.81	−5.64	−5.99	−5.29	−6.52	−4.75	CGE	[21]
Linepithema humile	0.000424	2.20E-06	2.38E-05	−5.66	−4.62	−4.90	−5.12	−4.69	−5.75	−4.06	CGE	[23]
Pogonomyrmex rugosus	0.0233	6.08E-06	2.43E-05	−5.22	−4.61	−4.55	−4.72	−4.38	−5.38	−3.72	DGE	[24]
Pogonomyrmex californicus	0.00676	1.69E-06	8.02E-06	−5.77	−5.10	−4.99	−5.23	−4.76	−5.84	−4.15	DGE	[21]
Pogonomyrmex occidentalis	0.00796	2.43E-06	1.96E-05	−5.61	−4.71	−4.87	−5.08	−4.66	−5.71	−4.03	DGE	[25]
Pogonomyrmex barbatus	0.0147	4.71E-06	1.86E-05	−5.33	−4.73	−4.64	−4.82	−4.46	−5.47	−3.81	CGE	[26]
Solenopsis xyloni	0.000431	2.10E-06	1.23E-05	−5.68	−4.91	−4.92	−5.14	−4.70	−5.76	−4.08	CGE	[23]
Incisitermes minor	0.0106	6.13E-06	2.95E-05	−5.21	−4.53	−4.55	−4.72	−4.38	−5.38	−3.72	Cyclic	[27]
Reticulitermes flavipes	0.00496	6.80E-06	1.59E-05	−5.17	−4.80	−4.51	−4.68	−4.35	−5.35	−3.68	Cyclic	[28]
Karoophasma biedouwensis	0.104	2.28E-05	2.57E-04	−4.64	−3.59	−4.10	−4.25	−3.95	−4.93	−3.27	Cyclic	[29]
Melanoplus sanguinipes	0.25	8.07E-05	2.24E-04	−4.09	−3.65	−3.66	−3.85	−3.48	−4.50	−2.83	CGE	[30]
Romalea guttata	2.92	7.88E-04	7.99E-04	−3.10	−3.10	−2.88	−3.19	−2.57	−3.75	−2.01	DGE	[31]

Species	Mass	Gas Uptake Rate (X)	Water Loss Rate (Y)	Log X	Log Y	Predict	Confidence Interval		Prediction Interval		Pattern	References
	g	Mol O_2 Day^{-1}	Mol H_2O Day^{-1}			Y	Min	Max	Min	Max		
Romalea guttata	2.87	5.13E-04	3.6E-04	−3.29	−3.44	−3.03	−3.31	−2.74	−3.89	−2.17	Cyclic	[32]
Taeniopoda eques	2.04	4.69E-04	3.33E-04	−3.33	−3.48	−3.06	−3.34	−2.78	−3.92	−2.20	Cyclic	[32]
Nauphoeta cinerea	0.675	1.66E-04	8.43E-05	−3.78	−4.07						DGE	This study
	0.808	2.67E-04	2.04E-04	−3.57	−3.69						CGE	This study
Zophobas morio	0.58	2.24E-04	2.39E-04	−3.65	−3.62						CGE	This study

References.

1. Woodman, J.D.; Cooper, P.D.; Haritos, V.S. Cyclic gas exchange in the giant burrowing cockroach, *Macropanesthia rhinoceros*: Effect of oxygen tension and temperature. *J. Insect Physiol.* **2007**, *53*, 497–504, doi:10.1016/j.jinsphys.2007.01.012.
2. Bartholomew, G.A.; Lighton, J.R.B. Ventilation and oxygen consumption during rest and locomotion in a tropical cockroach, *Blaberus giganteus*. *J. Exp. Biol.* **1985**, *118*, 449–454.
3. Marais, E.; Klok, C.J.; Terblanche, J.S.; Chown, S.L. Insect gas exchange patterns: A phylogenetic perspective. *J. Exp. Biol.* **2005**, *208*, 4495–4507, doi:10.1242/jeb.01928.
4. Groenewald, B.; Bazelet, C.S.; Potter, C.P.; Terblanche, J.S. Gas exchange patterns and water loss rates in the Table Mountain cockroach, *Aptera fusca* (Blattodea: Blaberidae). *J. Exp. Biol.* **2013**, *216*, 3844–3853, doi:10.1242/jeb.091199.
5. Bartrim, H.; Matthews, P.G.D.; Lemon, S.; White, C.R. Oxygen-induced plasticity in tracheal morphology and discontinuous gas exchange cycles in cockroaches *Nauphoeta cinerea*. *J. Comp. Physiol. B* **2014**, *184*, 977–990, doi:10.1007/s00360-014-0862-8.
6. Matthews, P.G.D.; White, C.R. Reversible brain inactivation induces discontinuous gas exchange in cockroaches. *J. Exp. Biol.* **2013**, *216*, 2012–2016, doi:10.1242/jeb.077479.
7. Gray, E.M.; Chown, S.L. Bias, precision and accuracy in the estimation of cuticular and respiratory water loss: A case study from a highly variable cockroach, *Perisphaeria* sp. *J. Insect Physiol.* **2008**, *54*, 169–179, doi:10.1016/j.jinsphys.2007.08.014.
8. Marais, E.; Chown, S.L. Repeatability of standard metabolic rate and gas exchange characteristics in a highly variable cockroach, *Perisphaeria* sp. *J. Exp. Biol.* **2003**, *206*, 4565–4574, doi:10.1242/jeb.00700.
9. Woodman, J.D.; Cooper, P.D.; Haritos, V.S. Neural regulation of discontinuous gas exchange in *Periplaneta americana*. *J. Insect Physiol.* **2008**, *54*, 472–480, doi:10.1016/j.jinsphys.2007.11.006.
10. Dingha, B.N.; Appel, A.G.; Eubanks, M.D. Discontinuous carbon dioxide release in the German cockroach, *Blattella germanica* (Dictyoptera: Blattellidae), and its effect on respiratory transpiration. *J. Insect Physiol.* **2005**, *51*, 825–836, doi:10.1016/j.jinsphys.2005.03.014.
11. Duncan, F.D.; Krasnov, B.; McMaster, M. Metabolic rate and respiratory gas-exchange patterns in tenebrionid beetles from the Negev Highlands, Israel. *J. Exp. Biol.* **2002**, *205*, 791–798.

12. Schilman, P.E.; Kaiser, A.; Lighton, J.R. Breathe softly, beetle: Continuous gas exchange, water loss and the role of the subelytral space in the tenebrionid beetle, *Eleodes obscura*. *J. Insect Physiol.* **2008**, *54*, 192–203, doi:10.1016/j.jinsphys.2007.09.001.
13. Duncan, F.D.; Dickman, C.R. Respiratory strategies of tenebrionid beetles in arid Australia: Does physiology beget nocturnality? *Physiol. Entomol.* **2009**, *34*, 52–60, doi:10.1111/j.1365-3032.2008.00651.x.
14. Duncan, F.D.; Dickman, C.R. Respiratory patterns and metabolism in tenebrionid and carabid beetles from the Simpson Desert, Australia. *Oecologia* **2001**, *129*, 509–517, doi:10.1007/s004420100772.
15. Lighton, J.R.B. Simultaneous measurement of oxygen uptake and carbon dioxide emission during discontinuous ventilation in the tok-tok beetle, *Psammodes striatus*. *J. Insect Physiol.* **1988**, *34*, 361–367, doi:10.1016/0022-1910(88)90104-7.
16. Lighton, J.R.B. Ventilation in Namib desert tenebrionid beetles: Mass scaling and evidence of a novel quantized flutter-phase. *J. Exp. Biol.* **1991**, *159*, 249–268.
17. Chown, S.L.; Holter, P. Discontinuous gas exchange cycles in *aphodius fossor* (Scarabaeidae): A test of hypotheses concerning origins and mechanisms. *J. Exp. Biol.* **2000**, *203*, 397–403.
18. Chown, S.L.; Davis, A.L. Discontinuous gas exchange and the significance of respiratory water loss in scarabaeine beetles. *J. Exp. Biol.* **2003**, *206*, 3547–3556, doi:10.1242/jeb.00603.
19. Duncan, F.D. The role of the subelytral cavity in water loss in the flightless dung beetle, Circellium bacchus (Coleoptera: Scarabaeinae). *Eur. J. Entomol.* **2002**, *99*, 253–258, doi:10.14411/eje.2002.034.
20. Bosch, M.; Chown, S.L.; Scholtz, C.H. Discontinuous gas exchange and water loss in the keratin beetle *Omorgus radula*: Further evidence against the water conservation hypothesis? *Physiol. Entomol.* **2000**, *25*, 309–314, doi:10.1046/j.1365-3032.2000.00197.x.
21. Lighton, J.R.B.; Schilman, P.E.; Holway, D.A. The hyperoxic switch: Assessing respiratory water loss rates in tracheate arthropods with continuous gas exchange. *J. Exp. Biol.* **2004**, *207*, 4463–4471, doi:10.1242/jeb.01284.
22. Lighton, J.R.B. Direct measurement of mass loss during discontinuous ventilation in two species of ants. *J. Exp. Biol.* **1992**, *173*, 289–293.
23. Schilman, P.E.; Lighton, J.R.B.; Holway, D.A. Respiratory and cuticular water loss in insects with continuous gas exchange: Comparison across five ant species. *J. Insect Physiol.* **2005**, *51*, 1295–1305, doi:10.1016/j.jinsphys.2005.07.008.
24. Lighton, J.R.B.; Garrigan, D.A.; Duncan, F.D.; Johnson, R.A. Spiracular control of respiratory water loss in female alates of the harvester ant *Pogonomyrmex rugosus*. *J. Exp. Biol.* **1993**, *179*, 233–244.
25. Quinlan, M.C.; Lighton, J.R.B. Respiratory physiology and water relations of three species of Pogonomyrmex harvester ants (Hymenoptera: Formicidae). *Physiol. Entomol.* **1999**, *24*, 293–302, doi:10.1046/j.1365-3032.1999.00140.x.
26. Gibbs, A.G.; Johnson, R.A. The role of discontinuous gas exchange in insects: The chthonic hypothesis does not hold water. *J. Exp. Biol.* **2004**, *207*, 3477–3482, doi:10.1242/jeb.01168.
27. Shelton, T.G.; Appel, A.G. Cyclic CO2 release and water loss in the western drywood termite (Isoptera: Kalotermitidae). *Ann. Entomol. Soc. Am.* **2000**, *93*, 1300–1307, doi:10.1603/0013-8746(2000)093[1300:ccrawl]2.0.co;2.
28. Shelton, T.G.; Appel, A.G. Cyclic CO2 release and water loss in alates of the eastern subterranean termite (Isoptera: Rhinotermitidae). *Ann. Entomol. Soc. Am.* **2001**, *94*, 420–426, doi:10.1603/0013-8746(2001)094[0420:ccrawl]2.0.co;2.
29. Chown, S.L.; Marais, E.; Picker, M.D.; Terblanche, J.S. Gas exchange characteristics, metabolic rate and water loss of the Heelwalker, *Karoophasma biedouwensis* (Mantophasmatodea: Austrophasmatidae). *J. Insect Physiol.* **2006**, *52*, 442–449, doi:10.1016/j.jinsphys.2005.12.004.

30. Rourke, B.C. Geographic and altitudinal variation in water balance and metabolic rate in a California grasshopper, *Melanoplus sanguinipes*. *J. Exp. Biol.* **2000**, *203*, 2699–2712.
31. Hadley, N.F.; Quinlan, M.C. Discontinuous carbon dioxide release in the eastern lubber grasshopper *Romalea guttata* and its effect on respiratory transpiration. *J. Exp. Biol.* **1993**, *177*, 169–180.
32. Quinlan, M.C.; Hadley, N.F. Gas exchange, ventilatory patterns, and water loss in two lubber grasshoppers: Quantifying cuticular and respiratory transpiration. *Physiol. Zool.* **1993**, *66*, 628–642, doi:10.1086/physzool.66.4.30163812.
